# Supplementary material for: Disruption of HaVipR1 confers Vip3Aa resistance in the moth crop pest Helicoverpa armigera
Source: PLoS Biol. 2025 May 29;23(5):e3003165. doi: 10.1371/journal.pbio.3003165 (PMC12121769; doi:10.1371/journal.pbio.3003165)
Supplement: S1 Text — Fig A: Schematic diagram and genotyping of the HaVip3R1 gene edited by CRISPR/Cas9. Description: (A) Schematic representation of the sgRNA-targeted site and sequences in exon 3 of HaVip3R1. (B) Indel mutations in the G1 larvae from single-pair families between G0 and SCD, highlighting the target sequences of the wild-type HaVip3R1 allele and the CRISPR/Cas9-induced mutations. (C) Representative chromatograms showing the 16-bp deletion in HaVip3R1 after CRISPR/Cas9 editing. Fig B: Sashimi plot showing splicing disruption in the HaVipR1 transcript in the Ha477-resistant line. Description: Sashimi plot illustrating the disruption of normal splicing in the HaVipR1 gene in the Ha477-resistant line, caused by a transposable element in intron 1. Data were obtained from pooled RNA-seq from susceptible and resistant H. armigera lines, showing abnormal splicing in the resistant line. Fig C: Expression analysis of 8 Vip3A-related genes from Spodoptera frugiperda in H. armigera mid-gut transcriptome. Description: Variance-stabilized expression values for eight Vip3A-related genes in susceptible and resistant allelic (Ha85 and Ha477) lines of H. armigera. Statistical significance was calculated using a Student t test, and p-values are provided for each comparison. Data available in S2 Data. Fig D: Spatio-transcriptome expression of HaVipR1 in H. armigera. Description: Expression values of HaVipR1 across different stages (L2, L3, and L4) and gut compartments (L5) in H. armigera. The data were obtained from the spatio-transcriptome dataset of H. armigera, including standard error for each condition. Fig E: Expression of the HaVipR1 homologue in Bombyx mori midgut. Description: Expression analysis of the HaVipR1 homologue in Bombyx mori midgut, using data from the SilkBase database. The homologous gene, LOC101735440, was found to have peak expression in the midgut. Data available in S4 Data. Fig F: Expression of HaVipR1 in Spodoptera frugiperda mid-gut and Sf9 cell lines. Descripti [file pbio.3003165.s001.docx]

**Supporting Information for**

Disruption of HaVipR1 confers Vip3Aa resistance in the moth crop pest *Helicoverpa armigera*

Andreas Bachler^1^*, Amanda Padovan^1^, Craig J. Anderson^2^ , Yiyun Wei^3^ , Yidong Wu^3^, Stephen Pearce^1^, Sharon Downes^4^ , Bill James^1^, Ashley E. Tessnow^5^ , Gregory A. Sword^5^, Michelle Williams^1^, Wee Tek Tay^1^, Karl H. J. Gordon^1^, Tom K. Walsh^1^

**Corresponding Author:** Andreas Bachler

**Email:** [Andy.Bachler@csiro.au](mailto:Andy.Bachler@csiro.au)

**This PDF file includes:**

Supporting Information Methods

Figures A to H

Tables A to E

References

Supporting Information Text

**Methods**

**Analysis of expression of HaVipR1 homologue in *Spodoptera frugiperda* mid-gut and Sf9 cell lines.**

The Short-Read Archive (SRA) browser was used to find RNA sequence data from either Sf9 cell lines or *S. frugiperda* mid-gut derived tissues. Seven mid-gut samples and 13 Sf9 samples (Table E) were downloaded and aligned to the *S. frugiperda* reference using Hisat2 with default parameters (GCF_023101765.2, “AGI-APGP_CSIRO_Sfru_2.0”). The homologue for HaVipR1 in *S. frugiperda* was found using mmseqs easy-rbh and was identified as LOC118272819 “thyroglobulin” gene. Overall gene expression was analysed across samples using DeSeq2 and the normalised read counts for the Sf *HaVipR1* homologue was derived using plotCounts.


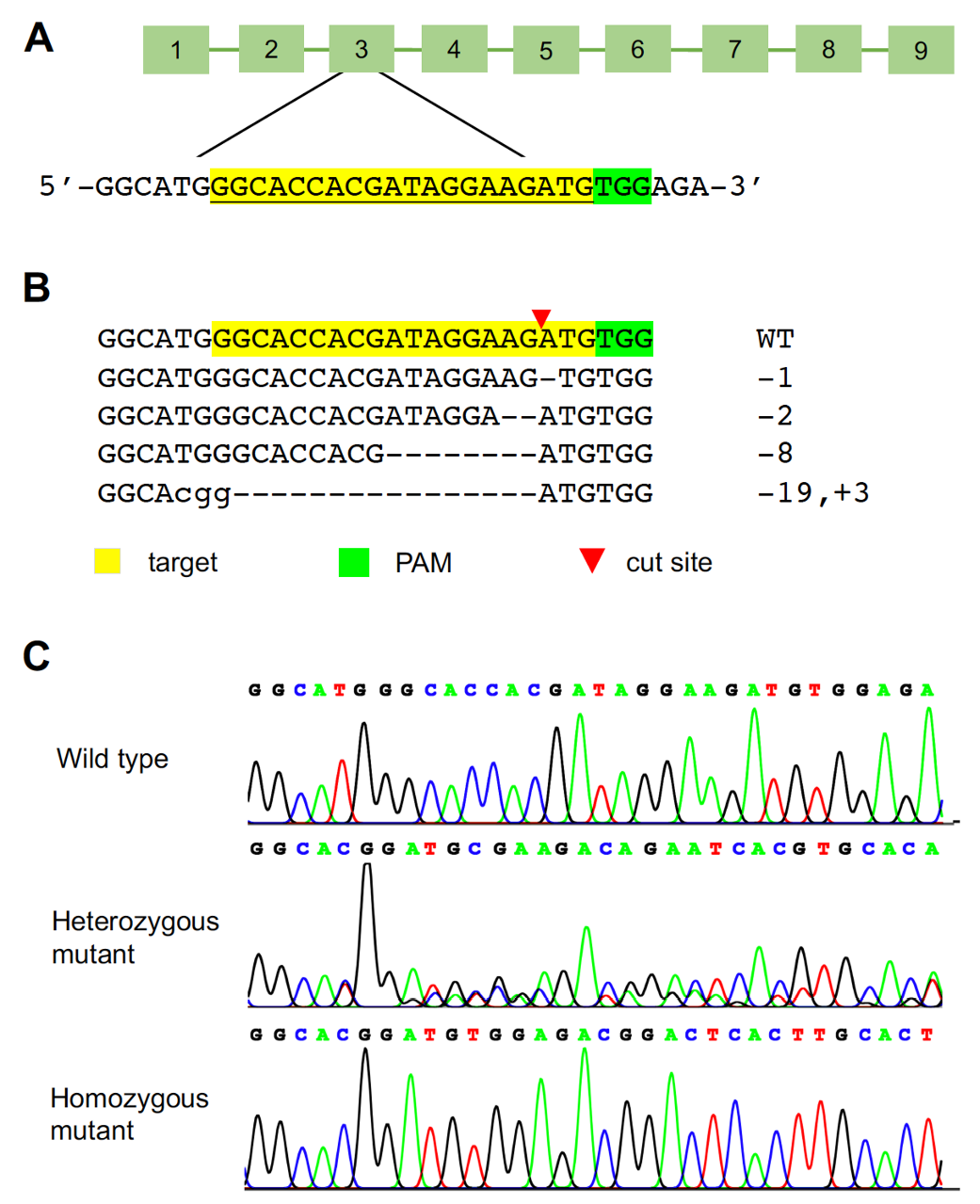


**Fig A.** (A). Schematic diagram of the sgRNA-targeted site and sequences in the exon 3 of HaVip3R1. (B). Sequences of indel mutations flanking the target site in the G1 larvae from the single-pair families between G0 and SCD. The target sequences of the wild type HaVip3R1 allele are highlighted in yellow and the PAM sequences in green. The cleavage site is indicated by a red triangle. Deleted bases are represented by dashes and inserted bases are shown in lower case. The numbers of bases deleted or inserted are listed at the right side of sequences. (C). Representative chromatograms of direct sequencing of PCR products for genotyping the 16-bp deletion of HaVip3R1 created by CRISPR/Cas9.


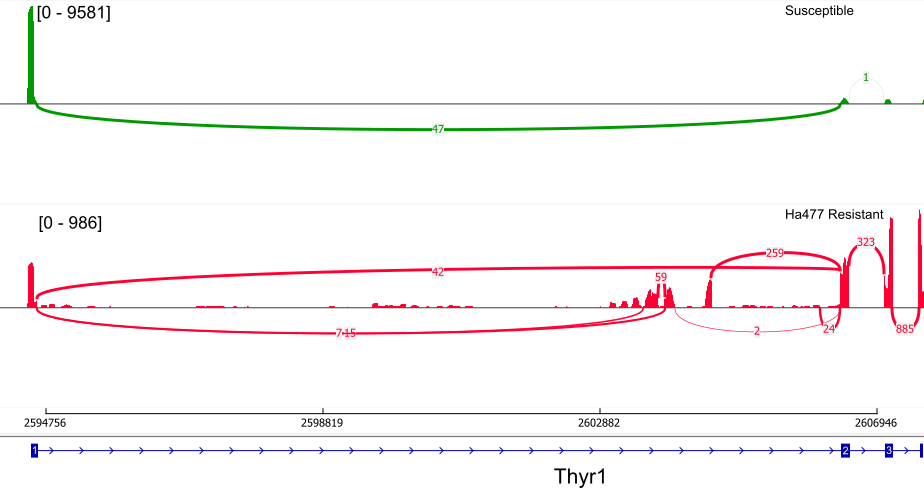


**Fig B. Sashimi plot demonstrating disruption of normal splicing of the *HaVipR1* transcript in the Ha477 resistant line**. Evaluation of the impact of the transposable element in intron 1 on the splicing of the HaVipR1 gene was conducted using genomic data from the Ha477 resistant line. Initially, a *de novo* assembled genome from an individual of the Ha477 resistant line was generated using long-read data and polished with matched short-read data. Subsequently pooled mid-gut RNA-seq data from three samples of the susceptible and resistant (Ha477) *H. armigera* lines were mapped to the genome assembly from an individual from the Ha477 resistant line. The *HaVipR1* gene coordinates were identified in the genome assembly using miniport and the first three coding exons are shown above. The resistant line displays abnormal splicing present from the first exon to a region which is repeat right near the end of the intron, and from this region there are also multiple spliced reads connecting to the second exon.There is no aberrant splicing is identified in the susceptible line.


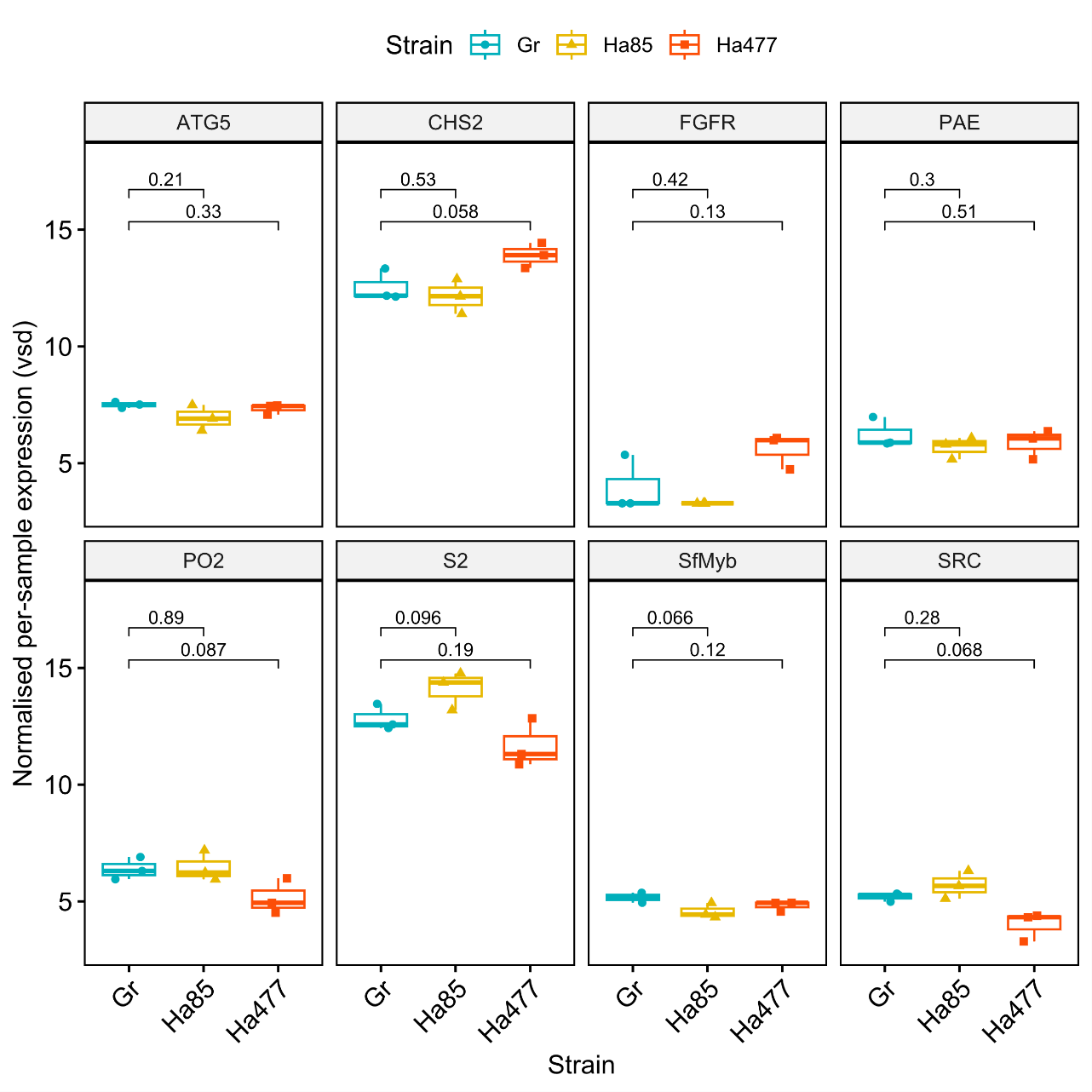


**Fig C. Analysis of expression of 8 other Vip3A related genes from *S. frugiperda* in pooled mid-gut transcriptome data from the susceptible and two resistant allelic (Ha85 and Ha477) lines of *H. armigera*.** Variance stabilised expression values are shown for each strain and statistical significance calculated using Students t-test for each of the resistant lines in comparison to the susceptible line. p-values are provided for each comparison. Data available in S2 Data.


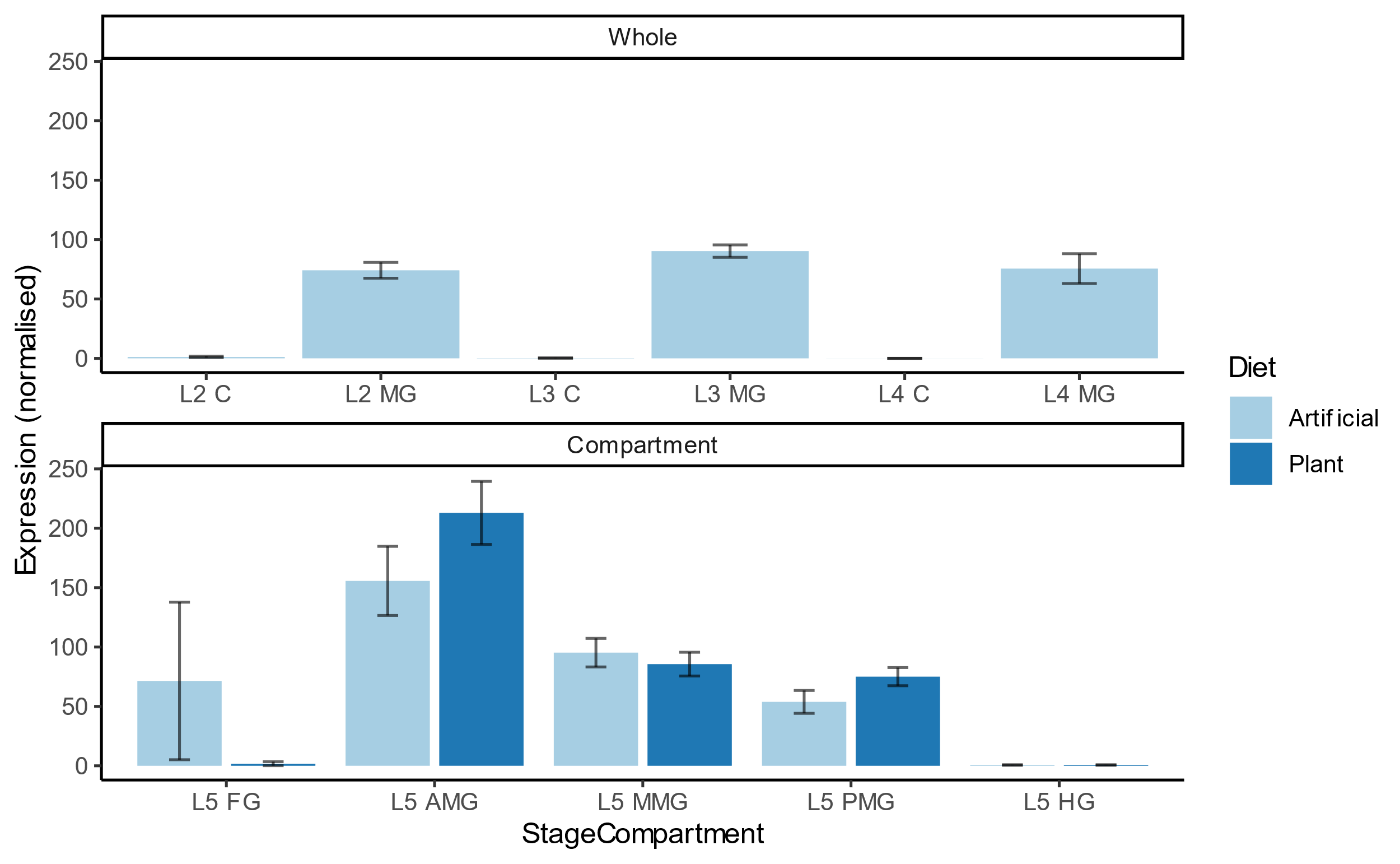
Fig D. Expression values for HaVipR1 (LOC110373801) in the spatio-transcriptome dataset generated from (1). Results for the whole body stages (L2, L3 and L4) and the compartment analysis (L5) have been shown along with the reported standard error. C=Carcass; MG=MidGut; FG=Foregut; AMG=Anterior MidGut; MMG=Middle MidGut; PMG=Posterior MidGut; HG=HindGut. Data available in S3 Data. For detailed developmental stage description please see Figure 1 from [1].


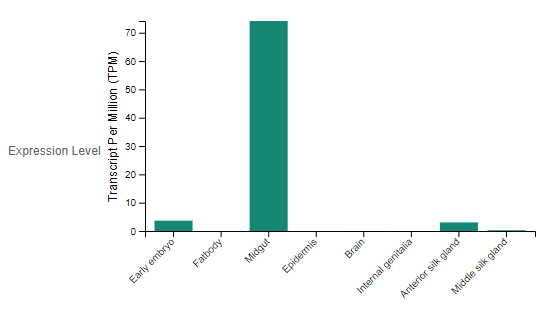


Fig E. Expression of the homologue of HaVipR1 in *Bombyx mori* indicates peak of expression of the gene in the midgut. The homologous gene for HaVipR1 in *Bombyx mori* was found to be LOC101735440 and this was used to search the SilkWorm database [2]. The matching record in the database is accession KWMTBOMO00841. The record can be accessed at this address https://silkbase.ab.a.u-tokyo.ac.jp/cgi-bin/entryview_gm2.cgi?clone_name=%20KWMTBOMO00841 . Raw values are provided in S4 Data.


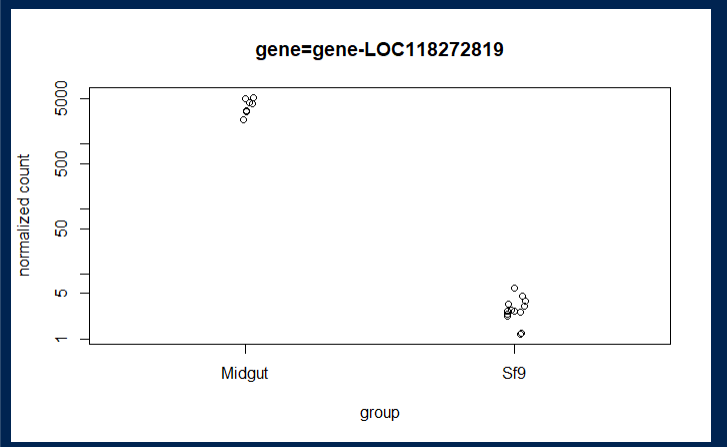


**Fig F. Expression of HaVipR1 homolog in *Spodoptera frugiperda* mid-gut tissues and Sf9 cell lines.** Samples analysed are described in Table D. Individual samples were aligned to the *S. frugiperda* reference and the normalised read counts for the homolog to HaVipR1 (LOC118272819) was extracted from DeSeq2. Data available in S5 Data.


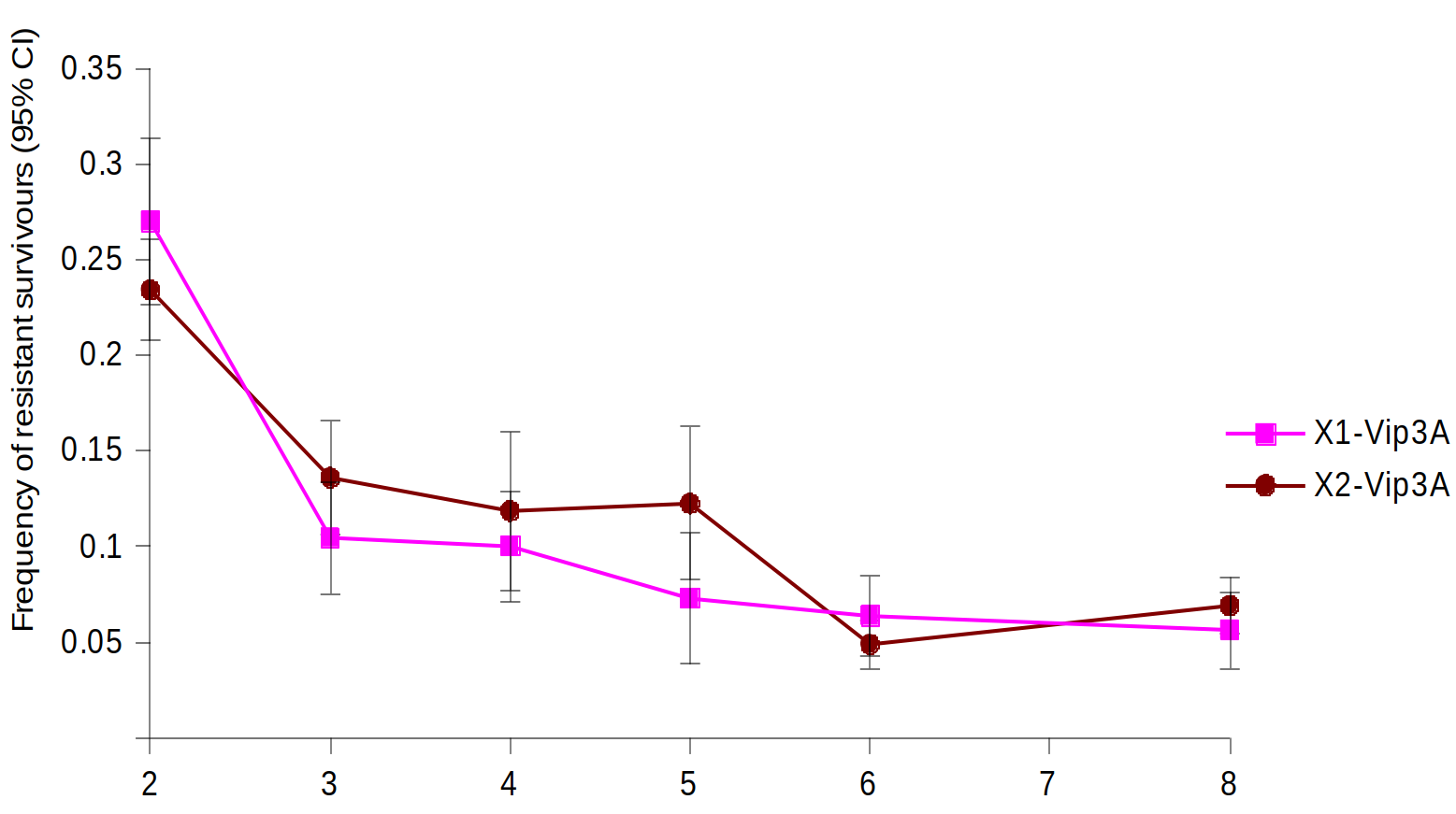
 Generation

**Fig G. In the absence of selection the HaVipR1 phenotype decreases over time.** The Vip3A resistant line Ha85 was maintained in the laboratory in the absence of selection for 8 generations and for both replicates (X1 and X2) the frequency of the Vip3A resistance phenotype declined over time. Data available in S6 Data.

**
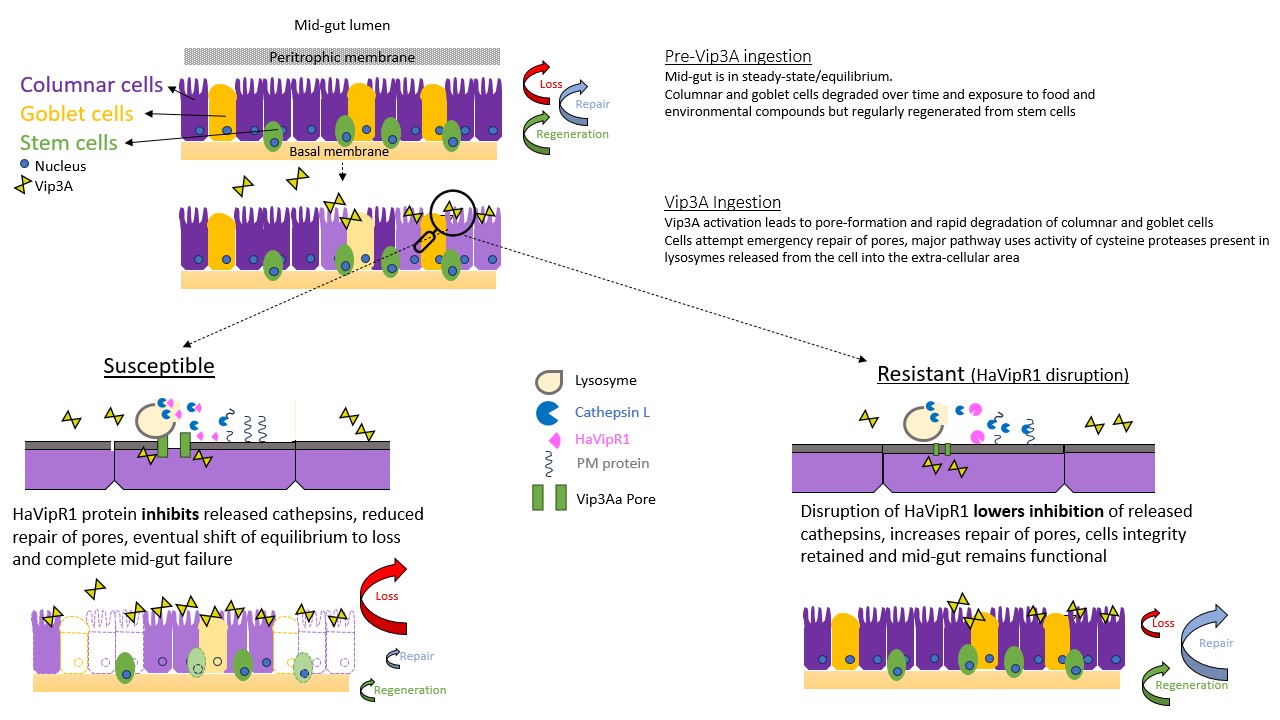
**

**Fig H. Proposed mechanism of Vip3Aa resistance through disruption of HaVipR1, based on its hypothesized role in mediating membrane repair in the midgut.**

**Table A. Number of RadTag loci per chromosome in female informative cross.**

| **Chromosome** | **Chromosome identifier** | **Number RadTag loci** |
| --- | --- | --- |
| *Z* | NC_064776.1 | 2,117 |
| *1* | NC_064777.1 | 423 |
| *2* | NC_064778.1 | 1,150 |
| *3* | NC_064779.1 | 1,094 |
| *4* | NC_064780.1 | 1,427 |
| *5* | NC_064781.1 | 1,250 |
| *6* | NC_064782.1 | 1,006 |
| *7* | NC_064783.1 | 1,237 |
| *8* | NC_064784.1 | 1,195 |
| *9* | NC_064785.1 | 1,451 |
| *10* | NC_064786.1 | 496 |
| *11* | NC_064787.1 | 1,289 |
| *12* | NC_064788.1 | 1,181 |
| *13* | NC_064789.1 | 1,021 |
| *14* | NC_064790.1 | 1,756 |
| *15* | NC_064791.1 | 1,072 |
| *16* | NC_064792.1 | 1,176 |
| *17* | NC_064793.1 | 1,188 |
| *18* | NC_064794.1 | 1,046 |
| *19* | NC_064795.1 | 866 |
| *20* | NC_064796.1 | 1,178 |
| *21* | NC_064797.1 | 1,005 |
| *22* | NC_064798.1 | 1,159 |
| *23* | NC_064799.1 | 530 |
| *24* | NC_064800.1 | 1,203 |
| *25* | NC_064801.1 | 713 |
| *26* | NC_064802.1 | 746 |
| *27* | NC_064803.1 | 921 |
| *28* | NC_064804.1 | 1,021 |
| *29* | NC_064805.1 | 438 |
| *30* | NC_064806.1 | 565 |

**Table B. Mapping statistics for transcriptome samples from the two resistant (Ha85 and Ha477) and susceptible (GR) *H. armigera* lines.**

| **Strain** | **Sample** | **Total Reads (counts)** | **Reads mapped (counts)** | **Reads mapped (%)** |
| --- | --- | --- | --- | --- |
| **Ha85** | S36 | 24,158,222 | 17,180,488 | 71.12% |
| **Ha85** | S39 | 24,129,320 | 17,913,549 | 74.24% |
| **Ha85** | S43 | 24,586,196 | 18,030,907 | 73.34% |
| **Ha477** | S15 | 26,006,796 | 18,047,080 | 69.39% |
| **Ha477** | S1 | 20,265,480 | 14,324,331 | 70.68% |
| **Ha477** | S28 | 29,702,064 | 20,037,062 | 67.46% |
| **GR** | S44 | 24,018,042 | 17,388,181 | 72.40% |
| **GR** | S45 | 24,405,054 | 17,526,844 | 71.82% |
| **GR** | S46 | 24,205,510 | 17,930,612 | 74.08% |

Table C. Read count and DeSeq2 statistics for the analysis of the HaVipR1 gene between the Ha85 resistant line and the GR susceptible line.

| Gene | baseMean | Log2FoldChange (LFC) | LFC  Standard Error | pvalue (raw) | pvalue (adjusted) |
| --- | --- | --- | --- | --- | --- |
| LOC110373801 | 1133 | -9.14 | 0.60 | 2.715 e^-52 | 1.844e^-48 |

Raw read counts for LOC110373801 gene from FeatureCounts:

Susceptible:

GR_1: 1923
 GR_2: 2902
 GR_3: 2081

Resistant:

Ha85_1: 0
 Ha85_2: 8
 Ha85_3: 4

Table D. Results from reciprocal best hit analysis of *S. frugiperda* (Sf) Vip3A related genes and *H. armigera* (Ha) genes.

| **SfGene** | **SfProt** | **SfGeneName** | **HaProt** | **HaGene** | **HaGeneName** | **Short name** |
| --- | --- | --- | --- | --- | --- | --- |
| LOC118275234 | XP_035449032.1 | Phenoloxidase subunit 2-like | XP_021182404.1 | LOC110370773 | phenoloxidase 1 | PO2 |
| LOC118279360 | XP_035454931.2 | Phenoloxidase- activating enzyme-like | XP_049706366.1 | LOC110371189 | phenoloxidase-activating enzyme | PAE |
| LOC118273105 | XP_050552797.1 | chitin synthase chs-2-like | XP_021184028.2 | LOC110371908 | chitin synthase chs-2-like | CHS2 |
| LOC118280749 | XP_035457001.2 | Scavenger receptor-C | XP_021184773.2 | LOC110372413 | MAM and LDL-receptor class A domain-containing protein 1 | SRC |
| LOC118277870 | XP_035452760.1 | Fibroblast growth factor receptor | XP_049693499.1 | LOC110373728 | fibroblast growth factor receptor homolog 1 | FGFR |
| LOC118262572 | XP_035429947.1 | Autophagy related gene 5 | XP_021187832.1 | LOC110374447 | autophagy protein 5 | ATG5 |
| LOC118263612 | XP_035431600.1 | Ribosomal S2 | XP_021191964.1 | LOC110377400 | 40S ribosomal protein S2 | S2 |
| LOC118279242 | XP_035454758.2 | myb protein | XP_049705840.1 | LOC126053489 | myb protein-like | SfMyb |

Table E. SRA identifiers from either Sf9 or *S. frugiperda* mid-gut samples analysed for HaVipR1 expression.

| **Sf9** | **Midgut** |
| --- | --- |
| SRR21707399 | SRR17042499 |
| SRR21707400 | SRR17042500 |
| SRR21707407 | SRR17042505 |
| SRR21707408 | SRR22019602 |
| SRR21707409 | SRR22019603 |
| SRR21707410 | SRR22019604 |
| SRR21707411 | SRR22019608 |
| SRR21707412 | |
| SRR21707413 | |
| SRR21707414 | |
| SRR21707415 | |
| SRR21707416 | |
| SRR21998727 | |

**References**

1. Ioannidis P, Buer B, Ilias A, Kaforou S, Aivaliotis M, Orfanoudaki G, et al. A spatiotemporal atlas of the lepidopteran pest *Helicoverpa armigera* midgut provides insights into nutrient processing and pH regulation. BMC Genomics. 2022;23(1):75. doi:10.1186/s12864-021-08274-x

2. Kawamoto M, Katsuma S. SilkBase Update: Addition of Information on Predicted Protein 3D Structures. Sanshi-Konchu Biotec. 2022;91(3):3_217–213_220. doi:10.11416/konchubiotec.91.3_217
