# Supplementary material for: Disruption of HaVipR1 confers Vip3Aa resistance in the moth crop pest Helicoverpa armigera
Source: PLoS Biol. 2025 May 29;23(5):e3003165. doi: 10.1371/journal.pbio.3003165 (PMC12121769; doi:10.1371/journal.pbio.3003165)
Supplement: Attachment — Submitted filename: PNAS_Main_ReviewerFeedback_Commented.docx [file pbio.3003165.s009.docx]

## Editor Remarks to Author:

We now have three thoughtful reviews of your manuscript. All three reviewers are complimentary regarding the multiple approaches that you have used to demonstrate the role of HaVipR1 in susceptibility/resistance to VIP toxins. However, each reviewer points out needs for additional data in order to have a more impactful study. Certainly, there is a need for dose-response data to show the extent of resistance due to lack of expression of HaVipR1 and the extent of resistance in heterozygotes --- is the resistance entirely recessive at all concentrations of the toxin?-- Perhaps this issue could be partially addressed by reference to previous studies, but I wonder if they would provide rigorous data related to just the resistance caused by lack of HaVipR1 expression.

Reviewers questioned the claim that identification of HaVipR1 offers insights into the mode of action of VIP toxins. As one reviewer points out, if the gene product "functions in some general detoxification/metabolic pathway (e.g. line 284), it is unlikely to reveal much about Vip3Aa mode of action." This takes away from the implication in your abstract. The same reviewer questions why resistance hasn't yet evolved, given that the null allele has been present and selected on for quite a few years. There could be a very interesting answer to this question, or it could be that the fitness cost to homozygotes is just too large. Appropriate control bioassays could address this issue. In rejecting your manuscript, I leave open the option for resubmission. If you choose that path, you must make changes to address each of the reviewers concerns or explain why specific concerns are not justified.

Reviewer Comments:

## Reviewer #1:

Suitable Quality?: No

Sufficient General Interest?: No

Conclusions Justified?: Yes

Clearly Written?: Yes

Procedures Described?: No

Comments:

In this study, Bachler and others used a combination of genomic, transcriptomic, and functional genetic approaches to identify the molecular mechanism underlying resistance to Vip3Aa in field-derived lines of Helicoverpa armigera. The field-derived lines, originally established in 2010, are interesting because they represent the H. armigera population prior to field deployment of Vip3Aa-expressing crops in Australia. Moreover, prior work (complementation tests) demonstrated that multiple individuals collected from the field over time for F1/F2 screens were resistant to Vip3Aa and had mutations in the gene described in their study. Resistance-conferring mutations in this gene persist at low frequency in Australian H. armigera populations today. Therefore, identifying the mutation that underlies resistance may provide insight into the Vip3Aa mode of action and/or resistance, which motivated the research.

To identify the molecular mechanism underlying resistance, the authors used RADSeq-enabled quantitative genomic analysis to identify chromosome 2 as bearing the mutation involved. They then narrowed in on the gene (with unknown function) involved using a transcriptomic analysis, which revealed strong differential expression of one gene. This gene was significantly downregulated in resistant lines, and the authors validated the involvement of this gene by generating CRISPR mutant lines lacking gene expression. Vip3Aa screening of a G2 population that was heterogeneous for the CRISPR mutation revealed that survivors were homozygous for CRISPR-mutant alleles. The authors then investigated the nature of the mutation in the original field-derived lines, revealing structural variants that resulted in impaired gene expression or splicing. They also compared the gene sequence with orthologs in other insects to identify its degree of conservation and analyzed the functional domains of this gene to better understand its potential functional role.

Overall, I find the work to be straightforward and it to be well-described (but see minor comment 1 below) and well done. I also find it to be important work. But where my opinion diverges from the authors is conclusions from and practical applications of the work. As written, the authors suggest that identification of this mutation may shed light on Vip3Aa mode of action and is useful for resistance monitoring. This may be the case under certain circumstances:

1. If this gene encodes a receptor for Vip3Aa, it might help to reveal the mode of action of Vip3Aa toxins. On the other hand, if it encodes a gene that functions in some general detoxification/metabolic pathway (e.g. line 284), it is unlikely to reveal much about Vip3Aa mode of action. It is not currently understood which is the case.

As described in our reply letter, we see that the framing of mechanism of action may not be appropriate given the lack of biochemical evidence of the function of *HaVipR1* and have updated the framing more toward ‘identification of a gene’, and provided some hypotheses for potential roles.

2. Mutations in this gene are already present in field populations of H. armigera, and have potential to be seized upon by selection, resulting in resistance. If indeed this happened, knowledge of this specific mutation would be very important. However, Vip3Aa-expressing crops have been commercially available in Australia since 2016 (line 128), and as of 2023, the mutation remains at low frequency (line 131).

Yes, these lines were from those characterised in the report of Vip3Aa resistance, and so pre-date introduction of Vip3Aa and field monitoring of Vip3Aa resistance remains low

My primary critique is that, as written, this work documents a mutation that may provide some insight into the Vip3Aa mode of action and may be important for Vip3Aa resistance monitoring of H. armigera. But it also may not, which detracts from the overall impact of the work and its broad appeal in the fields of resistance evolution and management. In my opinion, there is a larger story here which is perhaps being overlooked. One that, if told, may broaden the impacts of this work—both in terms of fundamental evolutionary biology and practical resistance management—making it appropriate to the broad readership of PNAS. A more interesting and broadly relevant set of questions to the agricultural community would be: why has this resistance-conferring mutation not been seized upon by selection, and what has prevented it from going to high frequency?

Currently we believe it is because of the adherence to Integrated Resistance Management (IRM) in Australia, with mandated Bollgard III + non-Bt refuges. Selection pressures require not only selection for Vip3Aa but also Cry1Ac and Cry2Ab simultaneously, all while maintaining fitness in the presence of selected populations in refuges, which appears sufficient for effectiveness of this pyramid crop at the moment.

Another way to frame the question would be to ask, under what conditions COULD it be seized upon by selection and result in resistance?

Potentially in cases of only Vip3Aa selection pressure, such as in single-expressing toxins in USA or Brazil? Characterization of disruptions of this gene would be useful as one target among many to monitor for resistance, which we feel provides clear value for this finding to be published

This is a fascinating empirical example of a large-effect mutation that is persisting in the H. armigera population but has not resulted in high levels of resistance, presumably due to resistance management practices or some other biological factor.

It seems the authors should be able to leverage their knowledge of the starting frequency of this mutation, its effect size and observed fitness cost (from this work), and the cropping systems (+ resistance management practices) it exists in to address these questions through modelling/simulation. As one of many potential example scenarios that could be addressed by modelling: if Vip3Aa were not introduced in a pyramid in Australian cropping systems, would this mutation have gone to high frequency?

We appreciate that a model of Vip3Aa with the evidence provided here could offer insights into the dynamics of selection. However, we believe the current understanding of pyramided Bt strategies and IRM aligns well with our findings:

1. Initial resistance allele frequencies for Vip3Aa were low before its introduction (Mahon et al. 2012).
2. There is a fitness cost associated with this resistance allele in the absence of selection (supplementary figure S7).
3. No cross-resistance exists between this allele and Cry1Ac or Cry2Ab (Chakroun et al. 2016).
4. Compliance with IRM strategies, such as refuges, is high in Australia (discussed by Knight et al. 2021).

The near-complete resistance to Vip3Aa from the disruption of HaVipR1 remains independent of survival against Cry1Ac or Cry2Ab. Mating between resistant individuals from selected populations and susceptible individuals from refuges dilutes the resistance allele frequency, provided that gene flow between populations remains. Selective pressures from Cry1Ac and Cry2Ab reduce the frequency of Vip3Aa resistance alleles, particularly in populations with high refuge compliance and low Cry resistance. Existing modelling studies have assessed Vip3Aa’s role in pyramided Bt systems under various scenarios, generally supporting these dynamics as long as Cry resistance remains low. For example, the study by Capiro et al. (2019)^[1]^ highlights the importance of low Cry resistance and adherence to IRM for maintaining Vip3Aa durability. F2 screening for resistance populations collected from the field in Australia confirm that Cry resistance remains low (Knight et al., 2021)^[2]^. While an explicit modelling approach may be useful, we feel that the description of the above and previous modelling work would mean we may not be contributing significantly with a model which reflects what is currently know. We propose that a focus on highlighting our experimental finds of a resistance gene as the major conclusion from this paper is suitable as it provides evidence supporting the theoretical framework already established by prior modeling studies. Future work could build on our findings by explicitly exploring and synthesising field data could provide a more comprehensive assessment than we can provide in the current work. We have included a discussion of our understanding of the background of this work as part of the Discussion (see lines 368-387).

Addressing such questions could provide interesting empirical support for the theoretical work done to develop resistance management strategies prior to Bt crop commercialization. It would provide practical resistance management insights for H. armigera, and more general, principles-level knowledge of why/how some mutations result in high levels of resistance in an insect species, while others do not. I think reframing the work in light of the above questions would be useful because, as it is currently written, the manuscript seems to inadvertently highlight one of the major issues with candidate gene monitoring. This is a gene candidate that should produce high levels of resistance in field populations of H. armigera, but it has not for the past 7–8 years. If effort were primarily expended on resistance monitoring of this gene, would it be possible to overlook other novel/unexpected Vip3Aa resistance mechanisms that lead to management failures?

We agree that just a focus on this one gene would be inadequate to fully capture Vip3Aa resistance dynamics. We currently have cases where there is Vip3Aa resistance detected in the field which is not allelic to a disruption in this gene. We would want efforts to be spent in both non-molecular (eg phenotypic F2 screens) as well as molecular monitoring, and disruptions of this gene, as well as other genes when they become characterised, would provide multiple lines of evidence for monitoring of Vip3Aa resistance, and to provide some targets for investigation of the effects of Vip3Aa in susceptible populations. We have attempted to clarify this position in the additional paragraph in the manuscript for lines 368-397.

A few minor concerns:

1. The transcriptomic experimental methods are not clearly described anywhere. One of the supplementary figures describes transcriptomic comparisons that I did not understand well from reading the text. I would strongly recommend that a brief description of the transcriptomic comparisons be added to the main text, and a longer, more thorough description of the experiments, data collection, and transcriptomic analysis be added to a "Methods" section in the supplement.

Thank you for the comment. I have re-written the methods section for the transcriptomic analysis and tried to clarify the results and interpretation in the main text

2. In reading the Discussion, I find it a bit challenging to understand the message the authors are trying to convey. Several paragraphs in the Discussion speculate on the potential role of this new gene in Vip3Aa resistance. I suggest that the authors consider adding a figure that describes their hypothesized potential roles of this gene in resistance, referenced alongside a clear in-text description of their hypotheses. Then the authors could support these mechanistic roles in subsequent paragraphs with examples/support from the literature.

I have added a diagram for the novel proposed hypothesis of HaVip3A in Vip3Aa resistance to supplementary materials (Figure S8)

3. Very minor - There appears to be a problem with the Fig. S3 caption label.

Apologies, this was some sample text from the PNAS template, fixed.

## Reviewer #2:

Suitable Quality?: Yes

Sufficient General Interest?: Yes

Conclusions Justified?: No

Clearly Written?: Yes

Procedures Described?: Yes

Comments:

This manuscript describes the identification of a novel gene, HaVipR1, whose mutations are linked to resistance to Vip3Aa in Helicoverpa armigera. By using genetic crosses and integrated genomic and transcriptomic analyses in an H. armigera population, Ha85, resistant to Vip3Aa and derived from field-collected individuals, a 149 bp deletion in the 3´ end of the HaVipR1 gene on chromosome 2 was shown to be linked to Vip3Aa resistance. In addition, a second H. armigera population resistant to Vip3Aa, Ha477, showed that resistance to Vip3Aa was linked to a transposon element insertion in the HaVipR1 gene. To confirm that mutations in the HaVipR1 gene confer resistance to Vip3Aa, a KO H. armigera mutant was constructed, showing that knocking out HaVipR1 confers resistance to Vip3Aa. The HaVipR1 gene was described as a novel, previously undescribed gene that is highly represented in different insect species. The analysis of the HaVipR1 gene suggested it encodes an extracellular protein probably involved in the inhibition of cysteine proteases, but its cellular function and the pathway leading to Vip3Aa resistance remain unknown. This is a novel gene associated with resistance to Vip3Aa, which could provide tools for resistance monitoring and also for fully understanding Vip3Aa's mode of action. Also, the conclusions are largely supported by the data.

However, in my opinion, the characterization of the H. armigera HaVipR1 KO is incomplete. Comparison of the resistance ratios of HaVipR1 KO with those of Ha85 and Ha477 is necessary to determine if other genes contribute to resistance in the field-derived populations.

Apologies for not providing this initially, provided now in manuscript.For both resistant lines and knockout strains there was less than 50% mortality for highest level of Vip3Aa surface toxin administered (lines 216-226). The knockout matches the Vip3Aa resistance phenotype identified in both of the field-derived Vip3Aa resistant lines.

In addition, cross-resistance to Cry1 and Cry2Ab toxins should be determined in the HaHaVipR1 KO. Finally, it is cited in the discussion that no defect on binding to Ha85 BBMV was reported before. Thus, binding analysis of Vip3Aa to HaVipR1 KO BBMV are necessary to conclude that HaVipR1 is not involved in binding of Vip3Aa to receptors.

The Australian Vip3Aa-resistant lines were previously characterized for cross-resistance to Cry1Ac and Cry2Ab as well as for changes in binding to Vip3Aa. Since the knockout line exhibited a high-level Vip3Aa resistance phenotype consistent with that of the resistant lines, we did not perform additional cross-resistance testing to Cry1Ac or Cry2Ab, nor did we conduct binding assays for the knockout line. We recognize, however, that this approach does leave open the possibility that the resistance mechanism in the knockout line could differ in some respects from that in the field-resistant line (similar to where the tetraspannin knock-in (SCD-KI) had a resistance ratio ~100x to Cry1Ac, while the back-crossed line (SCD423) had a resistance ratio ~1000x, indicating that there was some background mutations contributing to Cry1Ac resistance (Figure 3 in^[3]^)). In our current study, the knockout strain survived a higher exposure of Vip3Aa than was tested for the Australian resistant strains (500ug/cm2 for knockout vs 220ug/cm2 for Australian resistant lines) signifying that there is little indication that there are backgorund mutations contributing to resistance in this case. The original knockout line is no longer extant and so testing of resistance to Cry2Ab/Cry1Ac or BBMVs of the exact mutation identified in this manuscript is not possible. Yidong Wu has generated follow-up disruptions of this gene with the same ‘complete’ resistance phenotype, although these were with different mutations than the one currently described in this paper, and have been combined with other strains which means that testing of cross-resistance is not feasable for us currently.

Other points

Line 148. Describe the resistance ratio of the two Ha populations characterized in this work

Added results from previous work to text in manuscript, Results section lines 345-346

Line 222. Also, the analysis of transmembrane regions and GPI anchored sites in HaVipR1 should be analyzed and discussed

Added discussion of membrane domain in Discussion (320-333)

Line 219. Cross resistance of Vip3Aa resistance to Cry1A and Cry2Ab of the HaVipR1 KO

As discussed above.

Line 219. Provide the resistance ratio of the HaVipR1 KO

Apologies for omitting this, I have added the resistance ratio information to the Methods (lines 588-590) & Results (216-226) sections and as part of the Discussion (lines 345-347)

Line 219. Binding of Vip3Aa activated toxin to the HaVipR1 KO BBMV should be performed to rule out the possibility that HaVipR1 is not involved in receptor binding mechanism.

As discussed above, unable to be performed as KO line no longer extant.

Line 285. What are the resistance ratio for Vip3Aa protoxin compared to the activated toxin in the HaVipR1 KO? This could give a hint on a problem in processing the Vip3Aa protoxin in the resistant colonies

This is a good note. We conducted testing with proto-toxin exclusively to match field conditions, although changes between toxin and proto-toxin could indeed provide evidence to support or dismiss the hypothesis of changes in protease activity on toxin activation. However these arose from field monitoring which are proto-toxin exposed and may indicate that this mechanism is less likely to be able to be determined from changes from proto-toxin to toxin.

## Reviewer #3:

Suitable Quality?: Yes

Sufficient General Interest?: Yes

Conclusions Justified?: No

Clearly Written?: Yes

Procedures Described?: No

Comments:

In this manuscript, the authors identified a novel gene, HaVipR1, which was associated with resistance to Vip3Aa in the cotton bollworm through a combination of genetic crosses, genomic, and transcriptomic analyses. Furthermore, the role of HaVipR1 in Vip3Aa resistance was confirmed via CRISPR/Cas9-mediated knockout in the susceptible strain. Also, a short deletion in the 5' UTR and a large transposon element insertion in intron 1 of HaVipR1 were identified in the two tested Vip3Aa-resistant lines, respectively. Based on the functional annotation of HaVipR1, the authors speculated that HaVipR1 might play a distinct role in the mechanism of Vip3Aa resistance compared with previously reported Vip3Aa resistance genes. This identification of HaVipR1 could be helpful for the monitoring and management of Vip3Aa resistance in the field. However, there are several issues that need to be addressed to improve this manuscript.

Major issues
For both Vip3Aa-resistant lines and the HaVipR1-KO lines, there was no information about the levels of resistance (LC50 value or resistance ratio relative to the susceptible line) to Vip3Aa. A more detailed knowledge about the relative resistance levels of these lines is essential and would be useful to determine the relative role of HaVipR1 in the mode of action of Vip3Aa compared with other reported resistance genes.

Apologies for not including this the first time, added now in manuscript. Resistance of both the Australian resistant lines and the knockout is less than 50% for the highest tested Vip3Aa concentration. Lines 216-226

Although the role of HaVipR1 in Vip3Aa resistance was verified through CRISPR/Cas9-mediated knockout, no direct experimental evidences were provided in the manuscript to support that resistance to Vip3Aa in both Ha85 and Ha477 were caused by downregulation of HaVipR1 expression. The genetic complement tests of both resistant lines with HaVipR1-KO line need to be investigated to confirm whether the resistance locus in Ha85 and Ha477 were identical with that of HaVipR1 or not.

The two lines (Ha85 and Ha477) are complementary for Vip3Aa resistance (that is, F1 crosses of these two lines are resistant to Vip3Aa). Ha85 was back-crossed numerous times to reduce the contribution of background mutations to Vip3Aa resistance, leading to the single gene HaVipR1 being identified as disrupted. This was confirmed with qPCR, and Ha477 also shows reduced expression for this gene from qPCR evidence. Analysis of RNA-Seq data from Ha477 shows a different disruption of the HaVipR1 gene (from a transposable element which appears to disrupt normal splicing). We were not able to conduct complementation testing between the knockout and the Australian resistant lines as described in our reply letter, due to difficulties in moving the strains between continents (that is, moving a GMO line to Australia from China, or moving a known resistant line from China to Australia).

Line 271, how to explain the toxicity of Vip3Aa to Sf9 cell lines reported by (Jiang et al., 2023), if HaVipR1 "does not appear to be expressed in Sf9 cell lines".

We agree that this is challenging, however we note that there have been difficulties in translating findings of Vip3Aa toxicity from Sf9 cells to whole organisms (eg. Shan et al. 2021^[4]^.) We believe that the Sf9 cell line, while incredibly useful in some circumstances, might not be a suitable substitute for whole insects in general. Specifically, some of the findings of apoptosis or autophagy related to resistance to Vip3Aa have also been seen for other compounds, such as chemical pesticides azadirachtin (Shu et al. 2017^[5]^) or harmine (Shu et al. 2019^[6]^), and so potentially Sf9 cells might be providing insight into general response of (ovarian) cells to chemicals, and not specific to Vip3Aa. There are definite cases where Sf9 cells are an excellent, high-throughput screen for insecticidal compounds, but in cases where the insecticide might have very specific interactions (eg mid-gut) then the suitability of this model might be less relevant. Heterologous expression of HaVipR1 in Sf9, and a change in profile of Vip3Aa resistance, would be insightful and would be a progression available from the findings in this paper. The above is described in the Discussion (lines 279-291)

Minor issues

Line 93, a space between "with" and "Sf9" need to be added. There are several similar mistakes like above throughout the whole manuscript.

Apologies, looks like some kind of formatting error, tried to address throughout.

Line 243-245: How did the authors identify the 5' UTR of HaVipR1, and why can the removal of the Kozak sequence impact its expression?

Identification was from visual inspection of alignment of Ha85 long-read genomic data to the reference (added description of this in the Methods section). This was a follow-up after no other major impact variant was able to be identified from standard variant effect approaches. The deletion was immediately upstream of start codon and was flagged as ‘MODIFIER’ by SnpEff variant annotation. Added description of impact of disruption of Kozak sequence, this is generally on protein translation (Results lines 247-258 and Discussion 347- 352).

Supplementary Figure 1: Supplementary Figure 1 is hard to understand because the figure is confusing and the legend is unclear.

Apologies, re-worded and tried to clarify

Line 182: The FC of this gene should be far more than 10-fold.

Apologies, yes, looks like I'm referring to the log2(FC), not the actual fold change. I have tried to make this more consistent as part of the general methods update. Raw read count numbers and DeSeq2 summary stats provided in supplementary Table S5.

Line 177-179: Why were 500 reads selected as the threshold?

This was from a recommendation from an RNA-Seq data analysis course run by BioCommons Australia. I appreciate that it seems arbitrary, I have reduced it to 100 reads and re-generated the figures in the text, it didn’t noticeably change any of the main findings for Ha85. In checking another paper they do not describe their filtering in detail, but I believe this is done in general to reduce the impact of artefacts from genes with very low overall expression, where small fluctuations lead to large relative changes. (eg Benowitz et al. 2022^[7]^)

Line 198-199: The described information is not seen in Figure 4.

Apologies, mis-referenced from earlier version, fixed.

General Comment: The unit for expression values in the manuscript is confusing; please use a uniform unit throughout the article.

Apologies, should be consistent throughout and added raw numbers to allow for comparison in supplementary table S5

1. “The Corn–Cotton Agroecosystem in the Mid-Southern United States: What Insecticidal Event Pyramids Should be Used in Each Crop to Extend Vip3A Durability” <https://academic.oup.com/jee/article/112/6/2894/5543352>
2. “Successful development and implementation of a practical proactive resistance management plan for Bt cotton in Australia” <https://scijournals.onlinelibrary.wiley.com/doi/full/10.1002/ps.6490>
3. “Dominant point mutation in a tetraspanin gene associated with field-evolved resistance of cotton bollworm to transgenic Bt cotton” <https://pmc.ncbi.nlm.nih.gov/articles/PMC6243265/>
4. “Sf-FGFR and Sf-SR-C Are Not the Receptors for Vip3Aa to Exert Insecticidal Toxicity in Spodoptera frugiperda” <https://pmc.ncbi.nlm.nih.gov/articles/PMC9224979/>
5. “Transcriptome analysis of Spodoptera frugiperda Sf9 cells reveals putative apoptosis-related genes and a preliminary apoptosis mechanism induced by azadirachtin” <https://www.nature.com/articles/s41598-017-12713-9>
6. “Harmine induced apoptosis in Spodoptera frugiperda Sf9 cells by activating the endogenous apoptotic pathways and inhibiting DNA topoisomerase I activity” <https://pubmed.ncbi.nlm.nih.gov/30857624/>
7. “Novel genetic basis of resistance to Bt toxin Cry1Ac in *Helicoverpa zea*” [https://academic.oup.com/genetics/article/221/1/iyac037/6540856?login=true#352628165](https://academic.oup.com/genetics/article/221/1/iyac037/6540856?login=true#352628165)
