# Supplementary material for: Disruption of HaVipR1 confers Vip3Aa resistance in the moth crop pest Helicoverpa armigera
Source: PLoS Biol. 2025 May 29;23(5):e3003165. doi: 10.1371/journal.pbio.3003165 (PMC12121769; doi:10.1371/journal.pbio.3003165)
Supplement: Attachment — Submitted filename: Vip3Aa reply document Second Review.docx [file pbio.3003165.s010.docx]

The authors have addressed many of the previous editor’s and reviewers’ comments and clarified their methods, however a few important issues remain only partially resolved. They have not generated new dose-response data for heterozygotes to confirm whether resistance is recessive at all toxin concentrations, nor have they conducted cross-resistance or binding assays in the knockout line to confirm that HaVipR1-mediated resistance aligns with field-derived resistant lines. Although the original knockout line is no longer available for cross-resistance or binding experiments, characterizing heterozygotes remains essential.

*Thank you for your feedback. I appreciate your concerns regarding the complete characterization of heterozygotes and cross-resistance studies. I have followed up with our collaborator who conducted the original CRISPR knockout and can now confirm that dose-dependent resistance was assessed for heterozygous knockouts through F_1_ reciprocal crosses, which demonstrated that resistance was completely recessive. These new data have been incorporated into the manuscript [Results, lines 241-248; Methods, lines 631-640]*

*Regarding the alignment of resistance patterns between knockout and field-derived lines, both display near-complete resistance to Vip3Aa, with mortality not exceeding 50% at the highest testable concentrations (approximately 500 µg/cm² for the knockout line and 250 µg/cm² for field-derived lines). This robust resistance in the knockout line is particularly noteworthy given its completely susceptible SCD genetic background.*

*While we acknowledge the value of cross-resistance and binding studies with the knockout line, these experiments were not feasible due to significant logistical constraints. The knockout strain was generated in China (Yidong Wu's laboratory), while the resistant lines are maintained in Australia. Ideally we would have conducted complementary testing between the knockout and resistant lines, however this would require either importing the GMO knockout into Australia or transferring Vip3Aa-resistant lines to China - both options presenting considerable regulatory challenges and environmental risks. Given that binding studies and cross-resistance assessments were previously conducted with the field-derived lines, and that the knockout phenotype was identical, we believe the current data provide strong support for our conclusions while acknowledging these experimental limitations in the manuscript [Discussion, lines 368-371].*

It remains unclear whether HaVipR1 encodes a Vip3Aa receptor or performs a more general detoxification/metabolic role, and accordingly the authors have shifted their focus away from a definitive mechanism of action and toward identifying the gene and hypothesizing its possible roles. Nonetheless, they should moderate their claim that “HaVipR1-mediated resistance operates independently of known resistance genes, including midgut-specific chitin synthase and the transcription factor SfMyb.” While the evidence supports the possibility that HaVipR1 represents a novel mechanism of Vip3Aa resistance, the specific molecular pathway remains unknown, so this conclusion cannot be definitively established.

*Thank you for the careful reading. I have replaced that specific line with the more general: “Our research identifies HaVipR1 as a potential contributor to resistance, adding to our understanding of how insects may develop resistance to this important Bt protein.” I had initial feedback from my co-authors that the description of mechanism should be general and consider all options, including classic receptor mediated resistance, and feel that the section discussing this is generally moderate in it’s claims, although I have updated a few sentences to emphasise the need for further work (Discussion, 2 paragraphs, lines 347-405).*

While the authors suggest that integrated resistance management explains the low frequency of HaVipR1 mutations in the field, they do not supply new empirical or modeling data that directly test why HaVipR1 resistance alleles have not been favored by selection. The authors acknowledge that modeling and further field data could clarify why resistance alleles remain rare but believe existing theoretical and empirical work already supports the current observations. They emphasize that, while HaVipR1 is an important resistance gene, comprehensive monitoring, including other potential mechanisms, is crucial to managing Vip3Aa resistance. However, it is an overstatement to claim in the abstract that “identifying HaVipR1 forms a foundation for assessing resistance management strategies to preserve Bt crop efficacy.

*Thank you for the note. I agree with the summary here and affirm that in the Discussion we discuss the current understanding of resistance modelling around Vip3Aa resistance in the field [lines 408-427]. I have moderated the language relating to implications for resistance management throughout the manuscript and have removed the statement about HaVipR1 forming "a foundation for assessing resistance management strategies" and instead frame our findings more appropriately as contributing to the understanding of potential resistance mechanisms.*

*We feel that these changes better align our conclusions with the available evidence while maintaining the significance of identifying HaVipR1 as a gene associated with Vip3Aa resistance, the first in H. armigera, and one of only a handful of genes across Lepidoptera.*
