# Supplementary material for: Disruption of HaVipR1 confers Vip3Aa resistance in the moth crop pest Helicoverpa armigera
Source: PLoS Biol. 2025 May 29;23(5):e3003165. doi: 10.1371/journal.pbio.3003165 (PMC12121769; doi:10.1371/journal.pbio.3003165)
Supplement: Attachment — Submitted filename: Response to Academic Editor followup.docx [file pbio.3003165.s011.docx]

**Thank you for your thorough review of our manuscript. We appreciate the concerns raised regarding the characterization of resistance inheritance patterns and resistance ratios between our CRISPR knockout and field-derived lines. Below, we address these points in detail, providing additional context from the literature and clarifying our methodological choices.**

**Overall, we feel we can demonstrate that our current data robustly supports both the recessive inheritance of HaVipR1-mediated resistance and the comparable resistance levels between the CRISPR knockout and field-derived lines. The single-concentration inheritance testing aligns with established practices in the field, while our resistance ratio calculations at common concentrations provide quantitative support for our comparisons. We have updated the manuscript to better reflect these findings and their context within the broader literature on Bt resistance characterization.**

**1. The authors have not generated a full dose-response curve for heterozygous knockouts to confirm whether resistance is recessive at all toxin concentrations. While the newly added data indicate that resistance is recessive at 4 μg/cm², assessing a broader range of doses is necessary for a robust conclusion.**

Thank you for this concern. I would like to clarify how our current data addresses the inheritance pattern of HaVipR1-mediated resistance. In contrast to studies characterizing unknown genetic mechanisms of resistance where full dose-response curves are essential (e.g., Yang et al. 2021), our goal was specifically to confirm that the CRISPR-mediated knockout of HaVipR1 produces recessive resistance, similar to what has been described for the resistant lines Ha85 and Ha477.

Previous recent studies have demonstrated that inheritance patterns can be determined using single concentration points. For example, Jin et al. (2023) established recessive inheritance using a single discriminating concentration (0.4μg/cm²) for SfMyb-mediated resistance. Similarly, Bernardi et al. (2016) used two specific concentrations (2 and 3.6μg/cm², chosen to match previous field monitoring protocols, not to try and assess a range of values), with both concentrations supporting the same recessive inheritance pattern. In contrast, studies of lab-selected strains where the genetic basis is unknown, such as Liu et al. (2024), typically test multiple concentrations to characterize resistance evolution and potential background contributions. However, specifically for evaluating dominance from CRISPR-mediated gene disruptions, single concentrations are often used to test the F1 reciprocal crosses (see Zhang et al. (2024).)

In our case, testing heterozygotes at 4 μg/cm² provides evidence of recessive inheritance, as this concentration is below the established discriminating dose of 10 μg/cm² for *H. armigera* which we use to select between susceptible and resistant individuals. At this concentration, where partial resistance shoul be apparent, the heterozygotes behave exactly like the susceptible parent strains (that is, the resistant line survives completely, while no members of the susceptible or F1 lines survive).

We feel that this provides clear evidence that the disruption of the HaVipR1 gene in the SCD knockout is the primary contributor to the resistance seen against Vip3Aa. As none of the susceptible or F1 progeny survived at 4ug/cm2 assessing higher levels does not seem helpful. Testing lower levels of Vip3Aa will eventually lead to positive dominance values occurring, however this does not reflect the nature of inheritance of the genetic disruption, rather it reflects eventual tolerance of the concentration of toxin tested, as mentioned in Jin et al 2023 below.

*"... The concentration of Vip3Aa (in μg Vip3Aa per cm2 diet) at which dominance was evaluated was lower here (0.4) than in the three previous studies (3.6 or 10). Because the dominance of resistance to Vip3Aa increases as Bt toxin concentration decreases (26), the higher value of h seen here vs. previous studies could have been caused partly or entirely by the lower concentration of Vip3Aa used here."*

We would note that our level (4μg/cm2) feels appropriate in context of the resistance levels seen in other Lepidopteran pest species, like *Helicovpera zea* or *Spodoptera frugiperda*. In addition, previously there was mild background effects seen in F_1_ crosses of the Australian Ha85 lines with the susceptible line (~40% mortality seen at 4μg/cm2, Figure 2 Mahon et al 2012), while here we see that there is no survival at all in the F1 cross for the knockout line at this concentration.

I have updated the current manuscript to include a more thorough description of the reasoning for the single point concentration chosen and included the references above for context (lines 246-250).

**2. The authors could not directly compare the resistance ratios of the knockout line to those of the Ha85 and Ha477 lines, making it unclear whether additional genes beyond HaVipR1 might contribute to resistance in field-derived populations. The LC50 could not be established, as the maximum tested concentration (500 μg/cm² for the knockout and 220 μg/cm² for the resistant lines) resulted in less than 50% mortality. Thus, the claim that “the knockout matches the Vip3Aa resistance phenotype identified in both of the field-derived Vip3Aa resistant lines” remains unsupported by the current data.**

Thank you for this note. I would like to explain our approach to comparing resistance levels between the CRISPR knockout and field-derived lines, and clarify why we focused on reporting resistance at the highest concentration rather than resistance ratios.

Both the CRISPR knockout and field-derived resistant lines demonstrate what could be considered 'complete' resistance, with survival at concentrations far exceeding typical bioassay levels. While the maximum tested concentrations differ (500 μg/cm² for the knockout and 220 μg/cm² for the Australian resistant lines), both represent extraordinary levels of resistance. For context, a recent study in *Helicoverpa zea* (Yang et. al 2021) used a maximum concentration of 100 μg/cm² for resistance characterization, and when mortality didn't reach 50% at this level, they proceeded to calculate the resistance ratio using the highest tested level.

To enable direct comparison between our lines, I can use the highest common concentration of 220ug/cm2 tested in both the Australian resistant lines and the knockout lines. The resistance ratio calculations using available data show:

The LC50 for the susceptible Australian *H. armigera* strain is 0.551 (0.37–0.765) μg/cm² and the LC50 for the susceptible SCD strain used for the knockout is 0.32 (0.21-0.55) μg/cm². Using the maximum tested concentrations common to both (220ug/cm2) this yields resistance ratios of 660-fold for the Australian resistant lines and 687-fold for the CRISPR knockout line.

 I have included the calculation above in the updated manuscript (Results lines 234-244). I can’t seem to find the exact match for the sentence in my version of the current manuscript to update the wording? I see a similar sentence in the ‘*Summary*’ section and have updated the wording to be clearer here (lines 145-146).

**References**

Bernardi, O., Bernardi, D., Horikoshi, R.J., Okuma, D.M., Miraldo, L.L., Fatoretto, J., Medeiros, F.C., Burd, T. & Omoto, C. (2016). Selection and characterization of resistance to the Vip3Aa20 protein from Bacillus thuringiensis in *Spodoptera frugiperda*. Pest Management Science, 72(9), 1794-1802.

Jin, M., Shan, Y., Peng, Y., Wang, W., Zhang, H., Liu, K., Heckel, D.G., Wu, K., Tabashnik, B.E. & Xiao, Y. (2023). Downregulation of a transcription factor associated with resistance to Bt toxin Vip3Aa in the invasive fall armyworm. Proceedings of the National Academy of Sciences, 120(44), e2306932120.

Liu, Z., Liao, C., Zou, L., Jin, M., Shan, Y., Quan, Y., Yao, H., Zhang, L., Wang, P., Liu, Z., Wang, N., Li, A., Liu, K., Tabashnik, B.E., Heckel, D.G., Wu, K. & Xiao, Y. (2024). Retrotransposon-mediated disruption of a chitin synthase gene confers insect resistance to Bacillus thuringiensis Vip3Aa toxin. PLoS Biology, 22(7), e3002704.

Mahon, R.J., Downes, S.J. & James, B. (2012). Vip3A Resistance Alleles Exist at High Levels in Australian Targets before Release of Cotton Expressing This Toxin. PLoS ONE, 7(6), e39192.

Yang, F., Santiago González, J.C., Sword, G.A. & Kerns, D.L. (2021). Genetic basis of resistance to the Vip3Aa Bt protein in *Helicoverpa zea*. Pest Management Science, 77(4), 1530-1535.

Zhang, Z., Wang, L., Pang, X., Tay, W.T., Gordon, K.H.J., Walsh, T.K., Yang, Y. & Wu, Y. (2024). Knockout of the SfVipR1 gene confers high-level resistance to Bacillus thuringiensis Vip3Aa toxin in *Spodoptera frugiperda*. bioRxiv 2024.09.26.615236.
